# Virtual screening,docking and molecular dynamics simulation of selected phytochemical compounds bound to receptor tyrosine kinases:A correlative anti angiogenic study

**DOI:** 10.6026/97320630015613

**Published:** 2019-09-30

**Authors:** Garima Saxena, Salman Akhtar, Neha Sharma, Mala Sharma, M Haris Siddiqui, M Kalim A Khan

**Affiliations:** 1 Department of Bioengineering, Integral University, Lucknow, India; 2 Advanced Centre of Bioengineering and Bioinformatics, Integral Information and Research Centre, Integral University, Lucknow, India; 3 Department of Biosciences, Integral University, Lucknow, India; 4 Novel Global Community Educational Foundation7, Peterlee Place, Hebersham, NSW 2770, Australia

**Keywords:** Phytochemical, angiogenesis, anticancer, 1-Hydroxycryprochine, TIP database, molecular docking, molecular dynamics

## Abstract

Screening of phytochemicals for their anti angiogenic potential has been a growing area of research in the current decade. The following study proposes virtual screening, drug likeliness and ADME filtering of specific phytochemical based compounds retrieved from "TIP - A Database of Taiwan Indigenous Plants". The study further subjects the filtered phytochemicals for their molecular docking analysis and molecular dynamics simulation studies against the prominent receptor tyrosine kinases EGFR, VEGFR-1 and VEGFR-2 involved in angiogenesis phenomenon. Among the various in silico analysis done and precise interpretations, the current study finally proposes 1- Hydroxycryprochine as one of the most potent lead in combating angiogenic phenomenon and thus cancer. The following study involves all such important use of in silico platforms, tools and analysis protocols which are expected to reproduce commendable results in wet lab studies. The proposed compound 1-hydroxycryprochine tends to justify its anti angogenic potential in all interactional and stability studies.

## Background

Past researches report that there are multiple types of cancer; some of them provoke cells to grow and segregate at a moderate rate while others provoke swift cell and vessels growth. The latter uncontrolled growth of blood vessels from pre-existing one is termed as angiogenesis [1]. Normally,angiogenesis is a normal activity of growth and development in human body but it is also involved in wound healing and the generation of granulation tissue. In cancerous condition, chiefly it is an elementary step in the metamorphosis of tumors from a generous state to malignant. There are specific chemical signals in our body to initiate or regulate the angiogenesis phenomenon [1,2]. These chemical signals in the form of vascular endothelial growth factor (VEGF) and other epidermal growth factor (EGF), bind to receptors of the normal endothelial cells, and instigate the signals that enhance the growth and existence of new blood vessels from preexisting ones [3]. VEGF and EGF receptors are the receptor tyrosine kinase receptor (RTKs) and are found with very high propinquity on cell surface for multiple polypeptide growth factors. VEGF is a crucial signalling protein involved in angiogenesis, and can be further divided into 1, 2 and 3 with its respective functions. Similarly, EGFR is a trans-membrane protein which is initialized by the interaction of its inhibitors [3,4].

 Germinating angiogenesis is the first form of angiogenesis and appears in few well-distinguished phases. It involves mainly two stages; first, angiogenic growth factors which are known as biological signals initiate receptors on endothelial cells which are found on pre-existing blood vessels. In second stage, initialized endothelial cells start the secretion of enzymes proteases which degenerate the surface membrane so that it can permit endothelial cells to disappear from parent vessel membranes. Furthermore, the endothelial cells spread into its nearby matrix and produce rigid sprouts associating vessels and eventually commence angiogenesis[3].

Inhibition of angiogenesis phenomenon mainly involves the inhibition study of these prominent RTK’s (EGFR-1, VEGFR-1, VEGFR-2) through various mechanism which can thereby restrict the growth of new blood vessels [2]. There are multiple drugs available in the market against these proteins individually, duly approved by Food and Drug administration (FDA). EGF receptor protein inhibitory drugs include Gefitinib, Cetuximab, Neratinib, Erlotinib, Lapatinib, Osimertinib etc [5]. Similarly, VEGF receptors (VEGFR-1 and VEGFR-2) proteins inhibitory drugs include Foretinib, Brivanib, Lucitanib, Pazopanib and Staurosporine, Sorafenib, Axitinib, Sunitinib etc [2,6]. Though these drugs are being used in cancer treatment strategies but are found to be associated with prominent side effects and sometimes severe toxicity. In an attempt to overcome such adverse side effects associated with these drugs, several researchers have been carried out with specific focus on development of anti cancerous drug from natural phytochemical compounds [7,8,9]. Several phytochemical compounds obtained from Solanaceae, Crucifereae, Asteraceae families have been tested [10-14] and validated for their antiangiogenic potential [15]. The current study also focuses on inhibition study of selected phytochemical compounds against three RTKs (EGFR, VEGFR-1 and VEGFR-2) at the insilico platform which can be further validated in wet lab studies.

## Methodology

Various online and offline bioinformatics tool and software have been used in the successful accomplishment of the current study viz. PreADMET, AutoDock 4.2, Gromacs-4.0.5, Discovery Studio Visualizer, Chimera 1.12, and PRODRG.

## Proteins under study

The 3D structures of three prominent RTK's-EGFR, VEGFR-1 and VEGFR-2 were downloaded from PDB database with PDB ID's (4WKQ, 3HNG and 3VID). The selection of structural file was made on the basis of properties such as XRD resolutions, R-Value free/work and non-mutagenicity. The structures were cleaned and energy minimized using Chimera 1.12 to be used in further study. Selection and Retrieval of compounds from database In the current study, focusing on the immense potential of plant based compounds in anti cancerous research, a database of phytochemicals named 'TIP: A Database of Taiwan Indigenous Plants' was chosen and downloaded [16]. TIP consisted of 5,284 plant based compounds whose information along with their structures was downloaded in SD format.

## Virtual Screening

All 5,284 compounds of the TIP database were virtually screened for their drug-likeliness and pharmacokinetic properties using DS client 2.0. The purpose was to filter out the best compounds for further analysis in interactional studies. Christopher A. Lipinski had initially described the Lipinski's rule (Rule of five) to determine the drug likeliness of compounds by their pharmacokinetics, based on certain physical and chemical properties to make compounds orally active [17]. Though, this rule does not ascertain whether the compound is clinically active or not but Lipinski's rule do affirms these criteria for compound's credibility for future use [18].

## Toxicity Prediction:

Prediction of a drug molecule from lead compounds proceeds through multiple processes, in which few approaches give positive results and exceptionally approximately half of drugs fails due to limitation of ADME prediction in developmental phase. To check whether the filtered compounds are toxic or not, toxicity prediction studies were carried out on DS client. All standard inhibitors of target proteins along with filtered TIP database compounds were checked for toxicity. This prediction includes carcinogenicity, mutagenicity by Ames test and skin irritancy.

## Molecular Docking:

All the filtered compounds obtained from above study were subjected to molecular docking analysis with target proteins, in control with standard inhibitor using a standalone molecular docking suite AutoDock 4.2 [19,20]. Basically, docking analysis involve four major steps, they are as follows:

## Macromolecule Preparation:

Involves addition of polar hydrogen, removal of redundant water molecules and creating protein.pdbqt file of minimized receptor molecule

## Ligand Preparation:

Involves minimization and setting of torsion angles of ligands and saving them all in ligand.pdbqt file

## Grid Preparation:

Involves setting of a grid box around the active site of receptor molecule as a part of flexible docking and generating grid.gpf file

## Docking Parameters:

Involves setting of genetic algorithm runs and related parameters by creating a dock.dpf file

## Grid/Dock Run:

This final step involves the run analysis of grid.gpf and dock.dpf file generating grid.glg and dock.dlg file as final results respectively.

## Molecular Dynamics Simulation:

MD simulation is a computational approach, used to predict variability of molecules by making trajectories [21]. In the current study, the best posed bound complexes obtained from molecular docking study with highest binding energies were retrieved separately and subjected to MD simulation analysis using GROMACS 4.0.5. Before proceeding to MD simulation, selected compound's topology and gro file were generated on PRODRG (an online server), then further moving to dynamics GROMOS96 43a1 force field was applied to form protein ligand complex [21,22]. Further after addition of ions they were next subjected to energy minimization. NVT (volume regulation) and NPT (pressure regulation) was respectively run to complete the equilibration of system after energy minimization. Finally, 10 nanosecond MD run was applied with a leap-frog integrator of a step size of 2 fs in over all MD run and the results were saved at the interval of every 2 picosecond for stability analysis [21-23].

## Results and Discussion:

### Drug likeliness and ADME prediction

All 5,284 phytochemical compounds retrieved from 'TIP: A Database of Taiwan Indigenous Plants' database were virtually screened against Lipinski's rule (Rule of Five) and ADME (absorption, distribution, metabolism and excretion) filters using DS client. Lipinski's rule and ADME parameters focus on some vital atomic characteristics or activities to figure out pharmacokinetics of compounds which makes the compounds active orally. Some crucial parameters of Lipinski's rule and ADME are as explained below in Table 1.

On the basis of these parameters of Lipinski, all compounds were filtered on the basis of molecular descriptor calculator program. All calculated compounds were applied to another module named ADME descriptor for prediction of ADME properties. After application of these modules, total 494 phytochemicals were filtered out from 5,284 compounds on DS client and were further subjected to toxicity prediction analysis under the next step.

### Toxicity Prediction

All filtered 494 compounds along with standard inhibitors Gefitinib/Benzamide/Compound A for RTK's EGFR/VEGFR- 1/VEGFR-2 respectively were subjected to toxicity prediction analysis for their carcinogenicity, Ames test and skin irritancy parameters (Table 2).

In this study, the two best compounds filtered out from 494 compounds were numbered as TIP_1 (1-Hydroxycryprochine) and TIP_2 (Ethuliaconyzophenone). These two compounds tend to qualify all the rigorous Lipinski, ADME and toxicity prediction parameter in comparison to standard inhibitors and hence were finally subjected to further molecular docking analysis against RTK's under study.

### Molecular Docking

**Figure 1:**
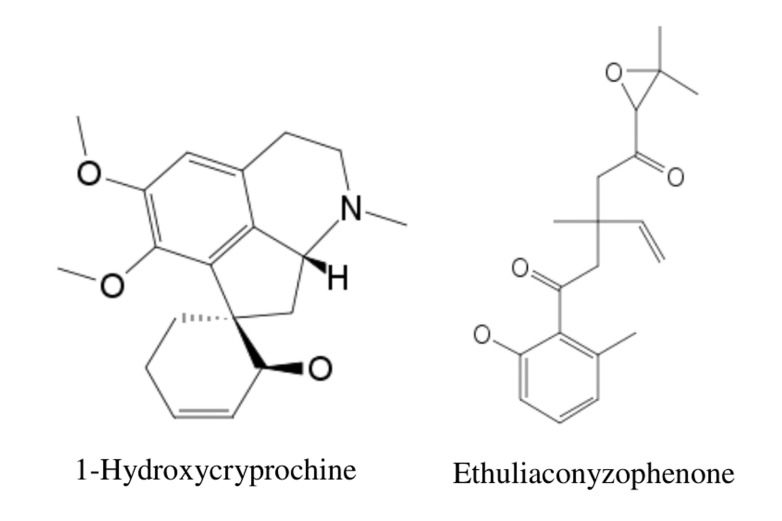
Structure of 1-Hydroxycryprochine (TIP_1) and Ethuliaconyzophenone (TIP_2)

To find best binding orientation of filtered compounds 1- Hydroxycryprochine and Ethuliaconyzophenone in control with standard inhibitors for RTK's under study, docking analysis was performed on AutoDock 4.2 with 25 genetic algorithm runs and all other default parameters. Flexible grid method was applied for docking to find best binding site in whole structure of all proteins. 1-Hydroxycryprochine and Ethuliaconyzophenone(Figure 1) were docked with EGFR, VEGFR-1 and VEGFR-2 separately to check their binding energies (BE) and inhibition constant (Ki).

Table 3 representing the docking results of all three proteins with 1-Hydroxycryprochine, Ethuliaconyzophenone and standard inhibitors clearly shows that 1-Hydroxycryprochine is in best binding energy mode amongst all standards with enhanced inhibition constant value while Ethuliaconyzophenone is showing comparatively lesser binding energy.

**Figure 2:**
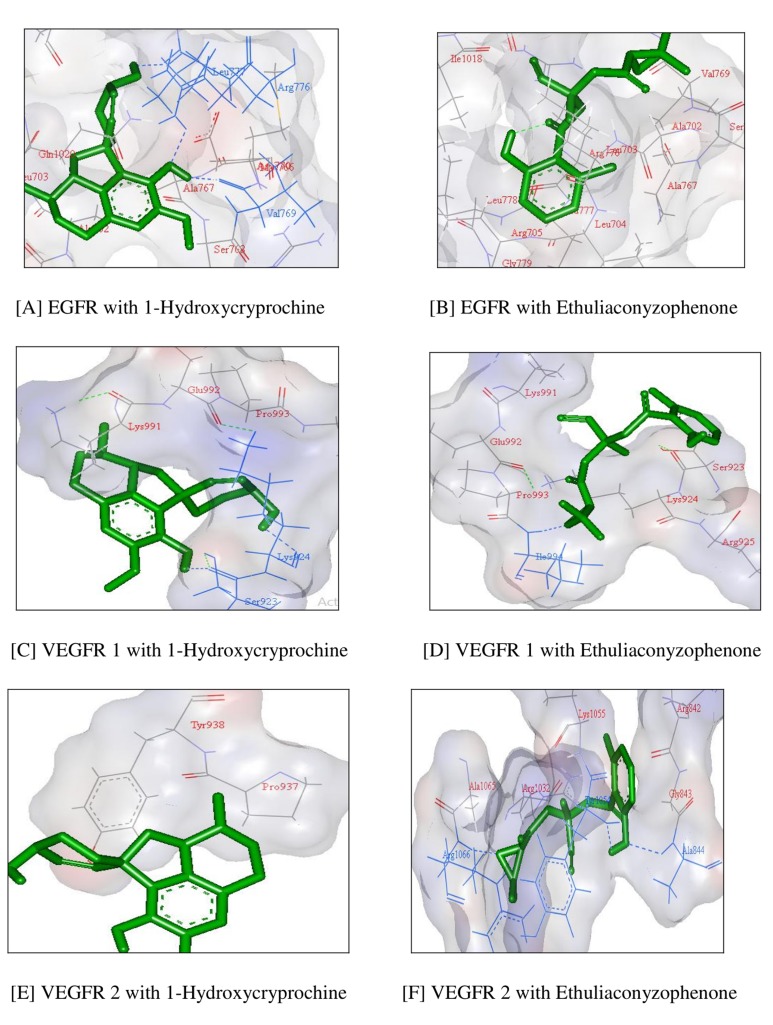
Docked complex structures of all three RTK's with1-Hydroxycryprochine and Ethuliaconyzophenone with H-bond representation.

Table 4 further suggests RTK EGFR in 3 H bonds interactions with 1-Hydroxycryprochine and no H bonds with Ethuliaconyzophenone as such. Similarly, VEGFR-1 is showing 2 and 1 H-bond with 1-Hydroxycryprochine and Ethuliaconyzophenone respectively and VEGFR-2 showing zero and 3 H-bond interactions with 1-Hydroxycryprochine and Ethuliaconyzophenone. The docking structures as visible in Figure 2 also revealed the similar type of interaction of these compounds with RTK's as like standard inhibitors, specifically in the active site pocket of proteins. Based on the above results, 1- Hydroxycryprochine was specifically selected to further MD simulation studies, for this compound showing better activity than Ethuliaconyzophenone and standard inhibitors in every aspect. 1- Hydroxycryprochine is a prominent pavine alkaloid isolated from the ethanol extract of the leaves of Cryptocarya chinensis [24]. C. chinensis (HANCE) HEMSL belonging to Lauraceae family is a widely distributed evergreen tree found in the low altitude forests in Taiwan and southern China. Researchers have reported this tree to contain many pavine and proaphorphine alkaloids where the pavine alkaloids have been specifically noted to possess various antiviral and immunological activities, behavioral and electrophysiological effects, and antiarrhythmic potential [24,25]. 1- Hydroxycryprochine, a pavine alkaloid has further been subjected here to in silico anti angiogenic assay and hence are carried forward to MD simulation studies with RTK's in the next step.

### Molecular Dynamics Simulation

**Figure 3:**
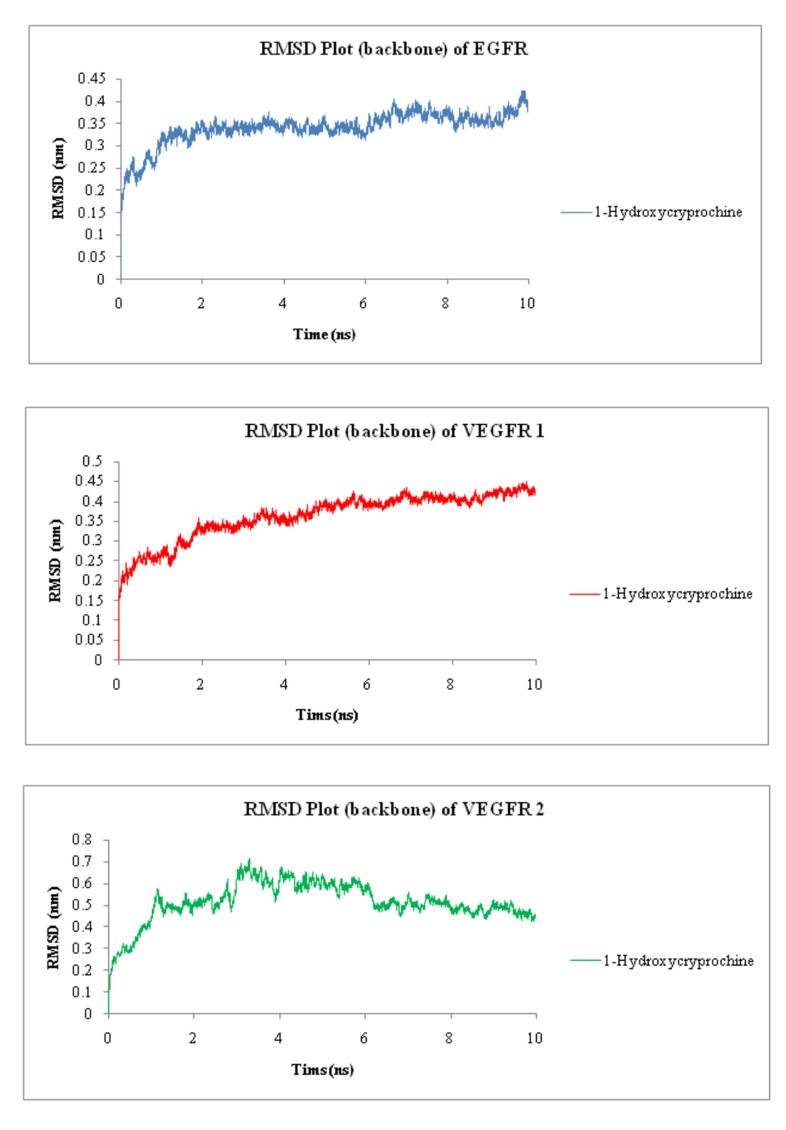
Root-Mean Square Deviation plot of backbone of proteins EGFR, VEGFR-1 and VEGFR-2 with 1-Hydroxycryprochine

Best docked results of 1-Hydroxycryprochine with EGFR, VEGFR-1 and VEGFR-2 were subjected to MD simulation studies on Gromacs- 4.0.5 modeling package using Linux as working platform. Prior to simulation, some initial files as gro and itp files of inhibitor 1- Hydroxycryprochine were prepared using PRODRG. Next GROMOS96 43a1 force field was applied followed by solvation, adding of ions, energy minimization and system equilibration (NVT and NPT) studies [21,22,23]. Lastly a 10 nanoseconds MD simulation was performed to build trajectories in analyzing the final results in the form of RMSD, RMSF and Radius of Gyration (Rg) (Figure 3).

Figure 3 clearly represents the graphical analysis of RMSD of all three proteins with 1-Hydroxycryprochine. In this study, plot [A] and [B] of EGFR and VEGFR-1 are showing more stability with 1- Hydroxycryprochine, while plot [C] of VEGFR-2 with 1- Hydroxycryprochine has been subject to some deviations.

**Figure 4:**
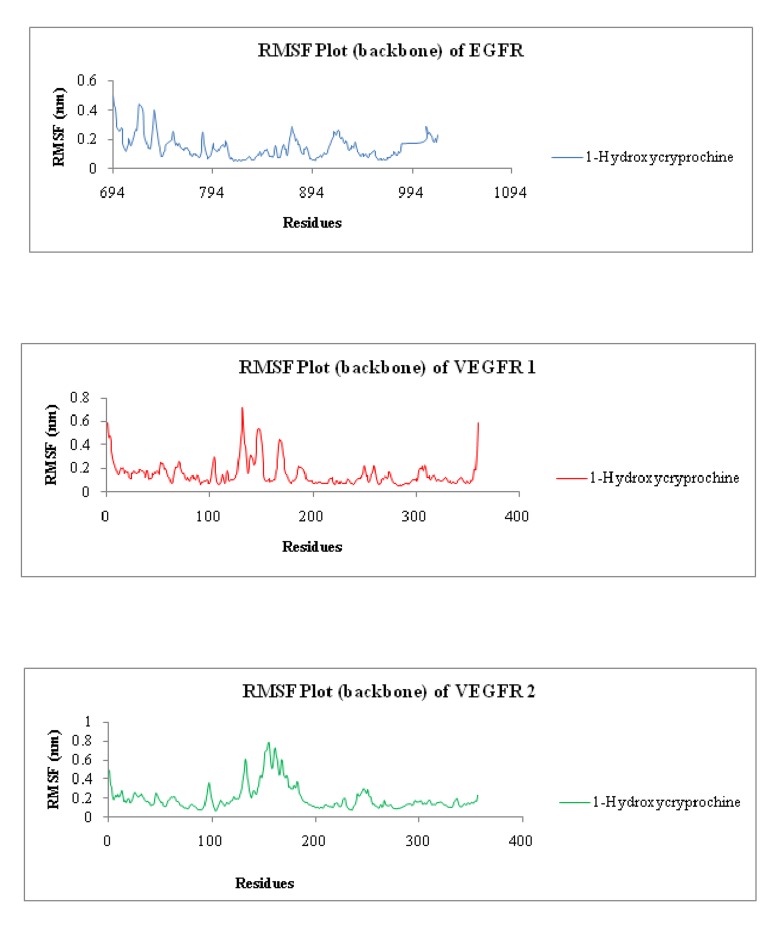
Root-Mean Square Fluctuation plot of backbone of proteins EGFR, VEGFR-1 and VEGFR-2 with 1-Hydroxycryprochine.

**Figure 5:**
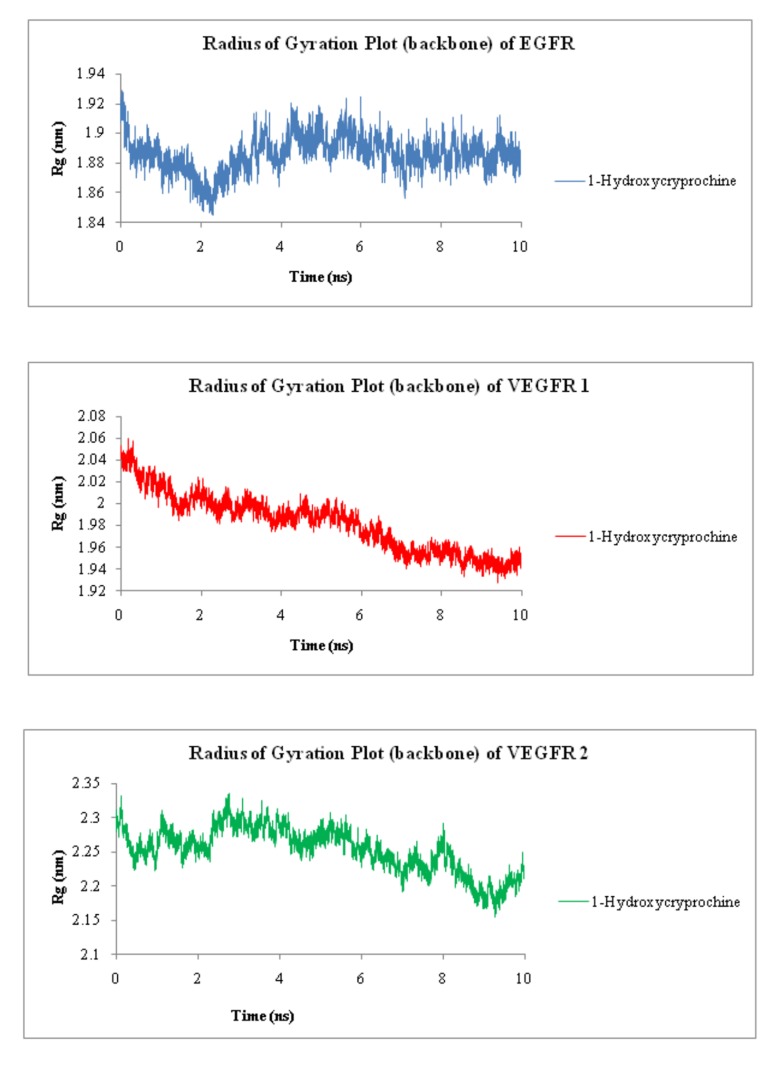
Radius of Gyration plot of backbone of proteins EGFR, VEGFR-1 and VEGFR-2 with 1-Hydroxycryprochine.

Similarly, Figure 4 is representing RMSF of all three proteins with1- Hydroxycryprochine separately. As we can clearly see that EGFR, VEGFR-1 and VEGFR-2 are showing fluctuation on some points of residues with 1-Hydroxycryprochine, they are showing very slight difference with the RMSF of same proteins as done with standard inhibitors. Likewise Figure 5 is representing radius of gyration plots of EGFR, VEGFR-1 and VEGFR-2 proteins with 1- Hydroxycryprochine respectively. These figures are clearly showing that all the three proteins with 1-Hydroxycryprochine are showing stability at some points of time, amongst which VEGFR-1 is showing best stability than VEGFR-2 and EGFR. Significantly, the compound 1-Hydroxycryprochine is been found in good interaction and stability conditions with all three proteins under study.

## Conclusion

The current study aimed to identify and retrieve a potent phytochemical compound having the ability to block the expression of RTK's EGFR, VEGFR-1 and VEGFR-2, thus combating the phenomenon of angiogenesis. In this process after virtual screening, ADME and toxicity filtering of 5,284 compounds of 'TIP' database, total two compounds (1-Hydroxycryprochine and Ethuliaconyzophenone) were selected for further docking and simulation studies. Subsequently, based on molecular docking analysis only 1-Hydroxycryprochine, a pavine alkaloid, was chosen in terms of best binding for MD studies Promisingly, 1- Hydroxycryprochine was seen to draw stable RMSD, RMSF and Rg trajectories with RTK's under study. Henceforth, the current study significantly proposes 1-Hydroxycryprochine obtained from C. chinensis Taiwanese tree as a promising phytochemical lead against the three targeted proteins which can be further validated under wet lab experiments in future studies.

## Figures and Tables

**Table 1:** Tabular representation of Lipinski's and ADME parameters

Lipinski's Rule		ADME	
Characteristics	Parameters	ADME Descriptors	Parameters
H-Bond Donor	<5	ADMET_BBB_level (Blood Brain Barrier)	<=2
H-Bond Acceptor	<10	ADMET_Solubility_level	<=3
Molecular Weight	<500 Da	ADMET_Absorption_level	<=1
miLogP	<5	ADMET_CYP2D6	0
-	-	ADMET_PPB_level (Plasma Protein Binding)	0
-	-	ADMET_Hepatotoxicity	0

**Table 2:** Toxicity prediction results of standard and best two compounds of the database

Compounds	Carcinogenicity	Ames Test	Skin Irritancy
Gefitinib	Non-Carcinogen	Non-Mutagen	Non-Irritant
Benzamide	Non-Carcinogen	Non-Mutagen	Non-Irritant
Compound A	Carcinogen	Non-Mutagen	Non-Irritant
TIP_1	Non-Carcinogen	Non-Mutagen	Non-Irritant
TIP_2	Non-Carcinogen	Non-Mutagen	Non-Irritant
1-Hydroxycryprochine (TIP_1) and Ethuliaconyzophenone (TIP_2)

**Table 3:** Binding energies (BE) and inhibition constant (Ki) of protein-ligand complex

Proteins	EGFR			VEGFR-1			VEGFR-2		
Compound	TIP_1	TIP_2	Gefitinib	TIP_1	TIP_2	Benzamide	TIP_1	TIP_2	Compound A
Binding Energy (kcal/mol)	-7.81	-5.75	-7.38	-8.76	-7.24	-8.63	-7.03	-6.3	-7.45
Inhibition constant (uM)	1.89	61.42	3.9	0.38	4.96	0.47	6.99	24.1	3.45
*TIP_1: 1-Hydroxycryprochine, *TIP_2: Ethuliaconyzophenone, Compound A: 4,5,6,11-tetrahydro-1H-pyrazolo[4',3':6,7]cyclohepta[1,2-b]indole

**Table 4:** Specific H-Bond interactions of 1-Hydroxycryprochine, Ethuliaconyzophenone with RTK's under study

Proteins	Compounds	H-Bond Interactions
EGFR	1-Hydroxycryprochine	A:ARG776:HH22-:UNK0:O21
		:UNK0:H42-A:VAL769:O
		:UNK0:H41-A:LEU777:O
	Ethuliaconyzophenone	NIL
VEGFR 1	1-Hydroxycryprochine	:UNK0:H42-A:SER923:O
		:UNK0:H41-A:LYS924:O
	Ethuliaconyzophenone	A:ILE994:H-:UNK0:O19
VEGFR 2	1-Hydroxycryprochine	NIL
	Ethuliaconyzophenone	A:ALA844:H-:UNK0:O9
		A:ARG1066:H-:UNK0:O19
		:UNK0:H27-A:TYR1054:N

## References

[1] Kim DW (2004). Curr Opin Investig Drugs.

[2] Hartmann JT (2009). Curr Drug Metab.

[3] Folkman JN (1971). Engl. J. Med.

[4] Hanahan D (1996). Cell.

[5] Moy B (2007). Nat RevDrug Discov.

[6] Akhtar S (2011). LettDrug Des Discov.

[7] Dias DA (2012). Metabolites.

[8] Mishra BB, Tiwari VK (2011). Eur. J. Med. Chem.

[9] Khan MKA (2015). J. Chem. Pharm. Res.

[10] Srivasatva R (2015). Bioinformation.

[11] Al Khodairy FM (2013). Am J Bioinfo Res.

[12] Akhtar S (2016). Interdiscip Sci Comput Life Sci.

[13] Sharma N (2018). Med Chem.

[14] Sharma N (2017). Med Chem.

[15] Arif JM (2013). Int J Bioinform Res Appl.

[16] Chun-Wei T (2014). Database: The Journal of Biological Databasesand Curation.

[17] Lipinski CA (1997). Adv.Drug Deliv Rev.

[18] Lipinski CA (2004). Drug Discov Today Technol.

[19] Morris GM (1998). J. Comput Chem.

[20] Morris GM (1996). J. Comput Aided Mol Des.

[21] Hess B (2008). J Chem Theory Comput.

[22] Bussi G (2007). J Chem Phys.

[23] Parrinello M (1981). J Appl Phys.

[24] Lin FW (2001). Chem Pharm Bull (Tokyo).

[25] Tian-Shung W, Fu-Wen L (2001). J Nat. Prod.

